# Supplementary material for: Unique Metabolomic Profile of Skeletal Muscle in Chronic Limb Threatening Ischemia
Source: J Clin Med. 2021 Feb 2;10(3):548. doi: 10.3390/jcm10030548 (PMC7867254; doi:10.3390/jcm10030548)
Supplement: Supplementary file 1 [file jcm-10-00548-s001.pdf]

Journal of
Clinical Medicine

MDPI

SUPPLEMENTARY MATERIALS

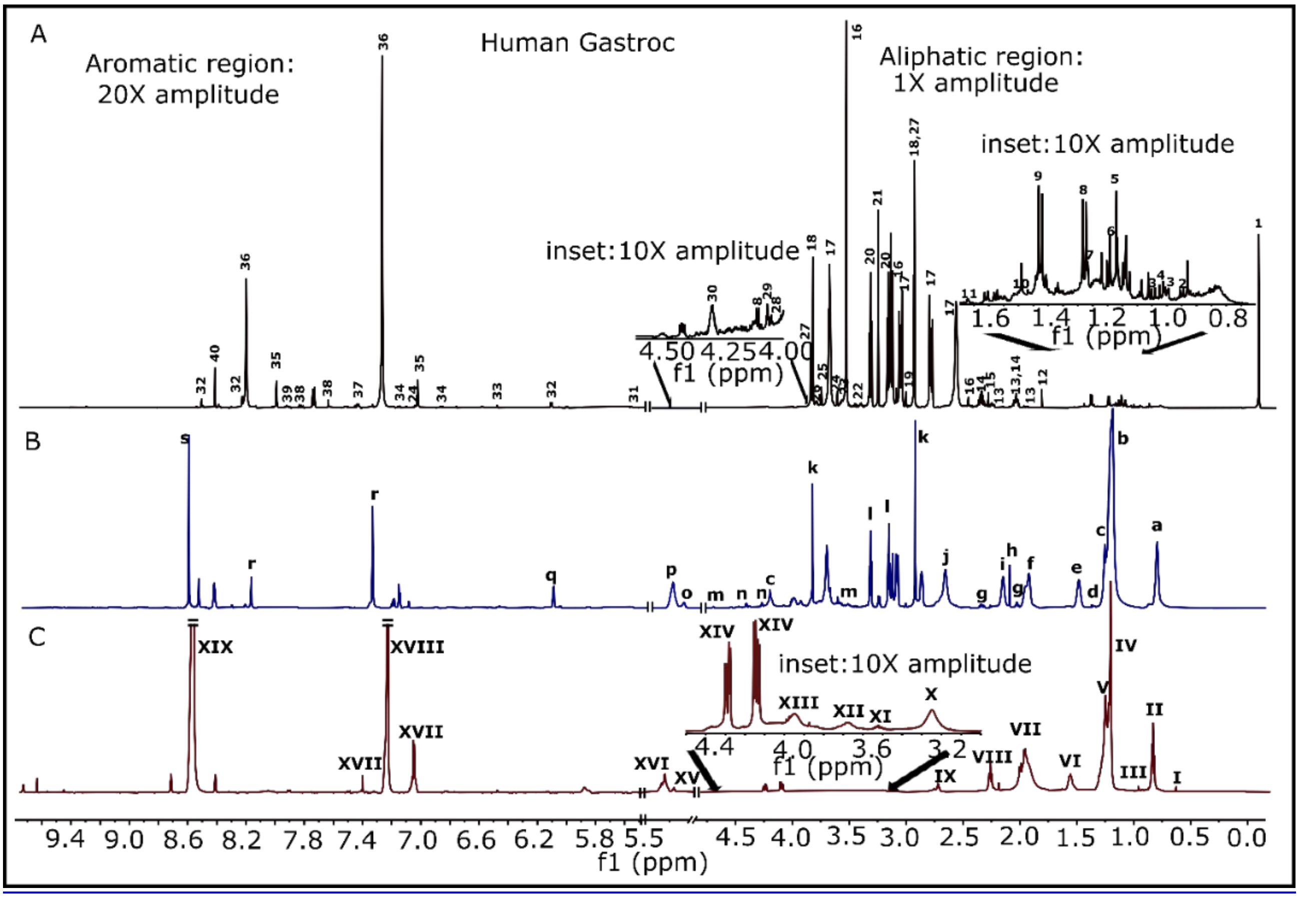

Figure 1. Comparison of NMR spectra taken for human gastrocnemius tissue samples using solution state NMR and HR-MAS. Water region (4.69–4.85 ppm) is removed. A) Solution state ^{1}H 1D NOESY spectrum for aqueous phase from FOLCH extraction, B) HR-MAS ^{1}H 1D NOESY spectrum, C) solution state ^{1}H 1D NOESY spectrum for organic phase from FOLCH extraction. Aromatic region is ~5-times magnified than aliphatic region and insets are ~10-times magnified.

In **Figure S1 A**: 1 represent DSS peak, 2 is leucine, 3 is valine, 4 is isoleucine, 5 is ethanol, 6 is 3-Hydroxybutyrate, 7 is threonine, 8 is lactate, 9 is alanine, 10 is lysine, 11 is arginine, 12 is acetate, 13 is glutamate, 14 is glutamine, 15 is 2-Aminoadipate, 16 is EDTA, 17 is MES buffer, 18 is creatine, 19 is malonate, 20 is taurine, 21 is methanol, 22 is glycerol, 23 is glycine, 24 is 3-methyl histidine, 25 is betaine, 26 is aspartate, 27 is creatine phosphate, 28 is O-phosphoethanolamine, 29 is myoinositol, 30 is Sn-Glycero-3-Phosphocholine, 31 is glucose, 32 is ATP/AMP, 33 is fumarate, 34 is tyrosine, 35 is Histidine, 36 is imidazole, 37 is phenylalanine, 38 is benzoate, 39 is tryptophan, and 40 is formate.

In **Figure S1 B**: a is CH_3-lipids + lipoproteins, b is $(CH_2)_n$ lipids + lipoproteins, c is lactate, d is alanine, e is ($\mathbf{CH_2}$-CH_2-CO-)lipids, f is (CH=CH-$\mathbf{CH_2}$-CO-)lipids, g is glutamine, h is succinate, i is (CH_2-$\mathbf{CH_2}$-CO) lipids, j is (=CH-$\mathbf{CH_2}$-CH=CH) lipids, k is creatine, l is taurine, m is glucose, n is CH_2OCOR (glyceryl), o is CH-glycerol, p is CH=CH lipids, q is ATP/AMP, r is imidazole, and s is pyrazine.

In **Figure S1 C**: I is (CH_3) cholesterol/cholesterol ester (C18) , II is (CH_3) cholesterol/esterified and free fatty acids, III is (CH_3) cholesterol (C19), IV is cholesterol, V is $(CH_2)_n$ of aliphatic chains, VI is ($\mathbf{CH_2}$-CH_2-COO-) β-methylene protons associated to carbon groups, VII is ($\mathbf{CH_2}$-CH=CH-CH_2) α-methylene protons associated to double bonds, VIII is ($\mathbf{CH_2}$-CCO-) α-methylene protons associated to carbonyl groups, IX is (=CH-$\mathbf{CH_2}$-CH=CH)divinyl methylene protons of w-3 and w-6 unsaturated fatty acids, X is $N^+(\mathbf{CH_3})_3$ in phosphadidylcholine, choline & sphingomyelin, XI is cholesterol, XII is glycerophospholipids, XIII is (3$\mathbf{CH_2}$-)glycerophospholipids, XIV is (-$\mathbf{CH_2}$-) triglyceride, XV is (**CH**-)triglyceride, XVI is (-**CH**=**CH**-) protons in double bonds in unsaturated fatty acids and –**CH** from cholesterol, XVII are Aromatic protons, XVIII is $CDCl_3$, and XIX is pyrazine.

Table S1. Chemical shift ranges with the correlated lipid classes that were responsible for the separation of ^{1}H-NMR metabolomics profiles in organic phase samples of PAD clinical models determined by PLSDA.

Spectra range (ppm)	Lipid class	Associated protons	Peak Pattern	VIP scores Organic phase (solution NMR)
2.03-2.07	Fatty acids /Triglyceride /Phopholipids	(–**CH**$_2$–HC=CH–CH$_2$)	m	~1.6
0.69-0.66/1.0-1.01	Cholesterol	C18/C19 **CH**$_3$	s	~1.6
5.18-5.23	Phospholipid	(**CH**–)	m	~1.6
0.78-0.92	Fatty acids/cholesterol/phospholipids	**CH**$_3$	m	~1.5
7.05-7.11	Aromatic protons	–**CH**	m	~1.3
5.29-5.42	Fatty acids	**CH**=**CH**	m	~1.2
2.03-2.07	Fatty acids /Triglyceride /Phopholipids	(–**CH**$_2$-HC=CH-CH$_2$)	m	~1.1
4.34-4.42	Triglyceride /Phopholipids	(1**CH**–)	m	~1.1
3.89-3.97	Glycerophospholipids	(3**CH**$_2$**–)**	m	~0.8
1.54-1.64	Phospholipid/Triglyceride	(**CH**$_2$–CH$_2$–COO)	m	~0.6
1.26-1.39	Fatty acids /Triglyceride /Phopholipids	(–**CH**$_2$)$_n$	m	~0.5
2.22-2.33	Triglyceride /Phopholipids	(**CH**$_2$–CCO–)	m	~0.4
4.26-4.32	Triglyceride	**CH**$_2$	dd	~0.4

Proton/s responsible for the corresponding peak/peaks in the spectra are shown with bold letters and peak pattern are represented as: 's' = singlet, 'd' = doublet, 'dd' = doublet of doublet, 't' = triplet, and 'm' = multiplet. VIP scores greater than 1 can be considered as the significant chemical shifts that are contributing mostly in driving separation among the three groups. Chemical shift with highest VIP score has been reported only for a particular metabolites/compounds. Bold font proton/s (in column 3) indicate the proton/s that is/are giving NMR peak/s at that particular spectral range (ppm).

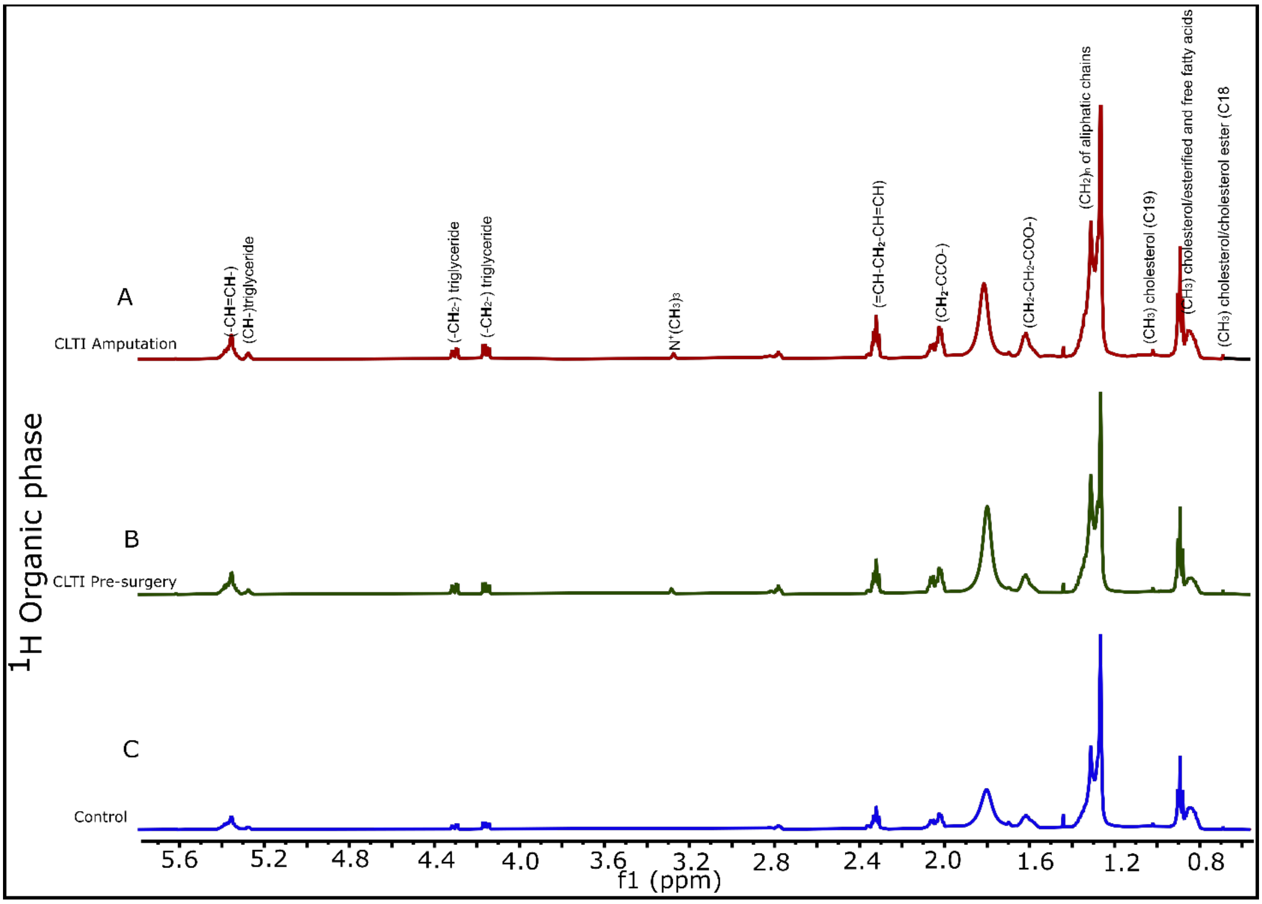

Figure S2. Representative ^{1}H NMR spectra for organic phase samples (normalized w.r.t. internal standard pyrazine peak at 8.61 ppm) for gastrocnemius tissues for all three groups: control, CLTI Pre-surgery, and CLTI Amputation. Samples

with similar wet weight were selected to make legitimate comparison. Figure S2A is CLTI Amputation#7a (10.7 mg), S2B is CLTI Pre-surgery#9a (10.5 mg), and S2C is control#8a (10.5 mg). Different lipid classes that are significantly varying among the three groups are assigned for convenience. CLTI Amputation (red), CLTI Pre-surgery (green), and control (blue).

Table 2. Average concentration (mM) or average peak intensity (A.U.) for all metabolites and/or compounds are reported for aqueous phase, organic phase, and HR-MAS data sets obtained with ^{1}H NMR and HR-MAS spectroscopy.

**Aqueous phase: ^{1}H NMR solution state**							
		**Average concentration (mM) ± S.D.**			***p*-value from ANOVA**		
**S.No.**	**Metabolite/s**	**Control**	**CLTI Pre-Surgery**	**CLTI Amputation**	**Control vs CLTI pre-Surgery**	**Control vs CLTI Amputation**	**CLTI Pre-surgery vs CLTI Amputation**
1	Leucine	0.033 ± 0.003	0.029 ± 0.008	0.084 ± 0.061	n.s.	0.049	n.s.
2	Valine	0.055 ± 0.006	0.046 ± 0.013	0.112 ± 0.071	n.s.	n.s.	0.042
3	Isoleucine	0.038 ± 0.003	0.037 ± 0.011	0.090 ± 0.063	n.s.	n.s.	n.s.
4	Isobutyrate	0.073 ± 0.023	0.095 ± 0.032	0.075 ± 0.026	n.s.	n.s.	n.s.
5	3-methyl-2-oxovalerate	0.064 ± 0.012	0.075 ± 0.020	0.067 ± 0.022	n.s.	n.s.	n.s.
6	Lactate	0.366 ± 0.110	0.243 ± 0.071	2.771 ± 2.498	n.s.	0.021	0.026
7	Alanine	0.364 ± 0.069	0.283 ± 0.089	0.663 ± 0.293	n.s.	0.019	0.006
8	Arginine	0.255 ± 0.083	0.277 ± 0.108	0.318 ± 0.121	n.s.	n.s.	n.s.
9	Lysine	0.189 ± 0.042	0.237 ± 0.083	0.190 ± 0.082	n.s.	n.s.	n.s.
10	Acetate	0.316 ± 0.223	0.473 ± 0.240	0.352 ± 0.193	n.s.	n.s.	n.s.
11	Glutamate	0.589 ± 0.180	0.478 ± 0.162	0.646 ± 0.247	n.s.	n.s.	n.s.
12	2-Aminoadipate	0.082 ± 0.015	0.099 ± 0.031	0.083 ± 0.032	n.s.	n.s.	n.s.
13	Pyruvate	0.308 ± 0.045	0.353 ± 0.105	0.321 ± 0.094	n.s.	n.s.	n.s.
14	Succinate	0.045 ± 0.009	0.044 ± 0.013	0.087 ± 0.039	n.s.	0.015	0.020
15	Glutamine	1.533 ± 0.550	0.791 ± 0.259	1.782 ± 0.831	n.s.	n.s.	0.027
16	Π-methyl histidine	0.198 ± 0.046	0.259 ± 0.050	0.141 ± 0.047	n.s.	n.s.	0.0006
17	Aspartate	0.205 ± 0.044	0.202 ± 0.065	0.281 ± 0.167	n.s.	n.s.	n.s.
18	Myo-inositol	0.106 ± 0.064	0.084 ± 0.050	0.188 ± 0.124	n.s.	n.s.	n.s.
19	Creatine	1.482 ± 0.210	1.050 ± 0.444	2.495 ± 1.337	n.s.	n.s.	0.022
20	Creatinine + PCr	0.894 ± 0.358	0.496 ± 0.240	0.708 ± 0.433	n.s.	n.s.	n.s.
21	Malonate	0.282 ± 0.044	0.330 ± 0610.	0.239 ± 0.076	n.s.	n.s.	0.043
22	Betaine	1.901 ± 0.444	2.486 ± 0.451	0.891 ± 0.553	n.s.	0.002	<0.0001
23	Taurine	4.321 ± 0.504	4.665 ± 0.790	3.504 ± 0.1.382	n.s.	n.s.	n.s.
24	Glycerol	0.367 ± 0.149	0.367 ± 0.105	0.653 ± 0.453	n.s.	n.s.	n.s.
25	Glycine	0.072 ± 0.027	0.058 ± 0.014	0.154 ± 0.070	n.s.	0.009	0.005
26	Sn-glycero-3-phosphocholine	0.046 ± 0.032	0.014 ± 0.010	0.062 ± 0.037	n.s.	n.s.	0.03
27	O-phosphocholine	0.217 ± 0.097	0.200 ± 0.133	0.267 ± 0.141	n.s.	n.s.	n.s.
28	Maltose	0.038 ± 0.022	0.027 ± 0.022	0.034 ± 0.021	n.s.	n.s.	n.s.
29	α-Glucose	0.005 ± 0.003	0.004 ± 0.003	0.163 ± 0.223	n.s.	n.s.	n.s.
30	Fumarate	0.003 ± 0.001	0.001 ± 0.000	0.008 ± 0.003	n.s.	<0.0001	<0.0001
31	Tyrosine	0.006 ± 0.006	0.000 ± 0.000	0.025 ± 0.025	n.s.	n.s.	0.03
32	Histidine	0.266 ± 0.189	0.010 ± 0.006	0.578 ± 0.571	n.s.	n.s.	0.04
33	Phenylalanine	0.036 ± 0.020	0.021 ± 0.007	0.104 ± 0.065	n.s.	0.015	0.006
34	Benzoate	0.168 ± 0.045	0.123 ± 0.045	0.177 ± 0.055	n.s.	n.s.	n.s.
35	Inosine	0.016 ± 0.010	0.011 ± 0.008	0.109 ± 0.071	n.s.	0.002	0.003
36	Formate	0.183 ± 0.065	0.248 ± 0.058	0.225 ± 0.084	n.s.	n.s.	n.s.
**Organic phase: ^{1}H NMR solution state**							
		**Average peak intensity (A.U.) ± S.D.**			***p*-value from ANOVA**		
**S.No.**	**Peak/Lipid component**	**Control**	**CLTI Pre-Surgery**	**CLTI Amputation**	**Control vs CLTI pre-Surgery**	**Control vs CLTI Amputation**	**CLTI Pre-surgery vs CLTI Amputation**
1	(CH_3) cholesterol/cholesterol ester (C18)	2.07 ± 0.37	2.69 ± 0.46	2.87 ± 0.54	n.s.	0.007	n.s.

2	(CH_3) cholesterol/esterified and free fatty acids	173.07 ± 73.11	407.86 ± 203.54	347.69 ± 157.62	0.03	n.s.	n.s.
3	(CH_3) cholesterol (C19)	4.19 ± 2.33	8.96 ± 5.17	9.48 ± 4.18	n.s.	0.04	n.s.
4	$(CH_2)_n$ of aliphatic chains	468.29 ± 125	981.33 ± 243.47	616.35 ± 241.81	0.001	n.s.	0.01
5	(C**H**$_2$–CH_2–COO–) β-methylene protons associated to carbon groups	80.60 ± 19.15	189.16 ± 75.64	122.39 ± 48.96	0.004	n.s.	n.s.
6	(C**H**$_2$–CH=CH–CH_2) α-methylene protons associated to double bonds	127.30 ± 58.17	217.80 ± 77.55	134.55 ± 41.96	0.034	n.s.	0.043
7	(C**H**$_2$–CCO–) α-methylene protons associated to carbonyl groups	54.83 ± 18.52	120.38 ± 29.29	69.61 ± 27.65	0.0006	n.s.	0.004
8	(=CH–C**H**$_2$–CH=CH)divinyl methylene protons of w-3 and w-6 unsaturated fatty acids	30.52 ± 6.11	46.77 ± 9.15	35.37 ± 12.17	0.023	n.s.	n.s.
9	N^+(C**H**$_3$)$_3$ in phosphadidylcholine, choline & sphingomyelin	10.70 ± 5.55	11.51 ± 5.00	12.57 ± 4.85	n.s.	n.s.	n.s.
10	(3C**H**$_2$–)glycerophospholipids	7.92 ± 3.07	5.98 ± 3.49	7.56 ± 2.06	n.s.	n.s.	n.s.
11	(–C**H**$_2$–) triglyceride	17.78 ± 6.03	39.02 ± 9.43	23.14 ± 9.21	0.0007	n.s.	0.007
12	1CH-Phospholipids + triglyceride	2.32 ± 1.08	2.91 ± 0.84	2.02 ± 1.09	n.s.	n.s.	n.s.
13	CH–Phospholipid	2.69 ± 1.05	2.59 ± 1.25	1.72 ± 1.04	n.s.	n.s.	n.s.
14	(C**H**–)triglyceride	7.53 ± 3.31	18.31 ± 4.81	9.61 ± 3.98	0.0004	n.s.	0.002
15	is (–C**H**=C**H**–) protons in double bonds in unsaturated fatty acids and –C**H** from cholesterol	68.79 ± 16.17	128.46 ± 26.51	80.64 ± 29.01	0.0012	n.s.	0.006

**^{1}H HR-MAS**							
		**Average peak intensity (A.U.) ± S.D.**			***p*-value from ANOVA**		
**S.No.**	**Peak/Lipid component**	**Control**	**CLTI Pre-Surgery**	**CLTI Amputation**	**Control vs CLTI pre-Surgery**	**Control vs CLTI Amputation**	**CLTI Pre-surgery vs CLTI Amputation**
1	Caprate	13084.27 ± 5838.28	9289.73 ± 5567.79	16813.36 ± 7402.78	n.s.	n.s.	n.s.
2	CH_3 lipid + lipoproteins	476287.61 ± 207308.17	686983.89 ± 354495.43	824058.86 ± 472699.46	n.s.	n.s.	n.s.
3	Leucine	23380.17 ± 9012.89	28135.30 ± 10057.48	36783.53 ± 15810.18	n.s.	n.s.	n.s.
4	CH_2 lipid + lipoproteins	2441401.41 ± 1081576.26	3548817.41 ± 2038398.91	4249379.56 ± 2455974.01	n.s.	n.s.	n.s.
5	Lactate	436870.58 ± 223482.27	667438.16 ± 246420.08	842161.32 ± 500851.47	n.s.	n.s.	n.s.
6	Alanine	4682.51 ± 3929.08	6949.01 ± 4286.98	6976.17 ± 3938.07	n.s.	n.s.	n.s.
7	(C**H**$_2$–CH_2–CO–)lipids	273081.04 ± 133411.54	417870.67 ± 213532.55	476485.56 ± 296909.26	n.s.	n.s.	n.s.
8	(CH=CH–C**H**$_2$–CO–)lipids	436670.36 ± 198080.60	642056.29 ± 313842.50	749793.64 ± 473916.53	n.s.	n.s.	n.s.
9	Glutamine	12255.62 ± 5874.45	6953.62 ± 3202.25	14244.43 ± 5105.60	n.s.	n.s.	n.s.
10	(CH_2–C**H**$_2$–CO) lipids	273380.60 ± 131030.24	408248.38 ± 207170.79	478095.76 ± 295189.96	n.s.	n.s.	n.s.

11	(=CH–**CH2**–CH=CH) lipids	251856.33 ± 90647.59	410418.68 ± 45427.77	175043.42 ± 122023.75	0.03	n.s.	0.0016
12	Creatine	43087.17 ± 8570.69	39659.88 ± 8258.93	42890.81 ± 18995.70	n.s.	n.s.	n.s.
13	Taurine	61142.46 ± 15723.28	70382.61 ± 7932.46	46485.58 ± 22315.84	n.s.	n.s.	n.s.
14	Glucose	2608.77 ± 1145.73	2622.30 ± 1180.86	2179.61 ± 1920.82	n.s.	n.s.	n.s.
15	CH2OCOR (glyceryl)	93204.00 ± 39588.85	126402.36 ± 58312.31	153033.57 ± 87731.77	n.s.	n.s.	n.s.
16	CH–glycerol	43204.39 ± 20189.11	64778.92 ± 31150.94	77811.03 ± 48679.94	n.s.	n.s.	n.s.
17	CH=CH lipids	267460.68 ± 129833.12	403227.65 ± 198230.93	473309.94 ± 310343.17	n.s.	n.s.	n.s.

Data are presented as mean ± S.D. One way ANOVA using Tukey's multiple comparisons test was performed on each metabolite/compounds and the p-values are also reported for each of them. Bold font proton/s indicate the proton/s that is/are giving NMR peak/s at that particular spectral range (ppm). n.s., not significant.

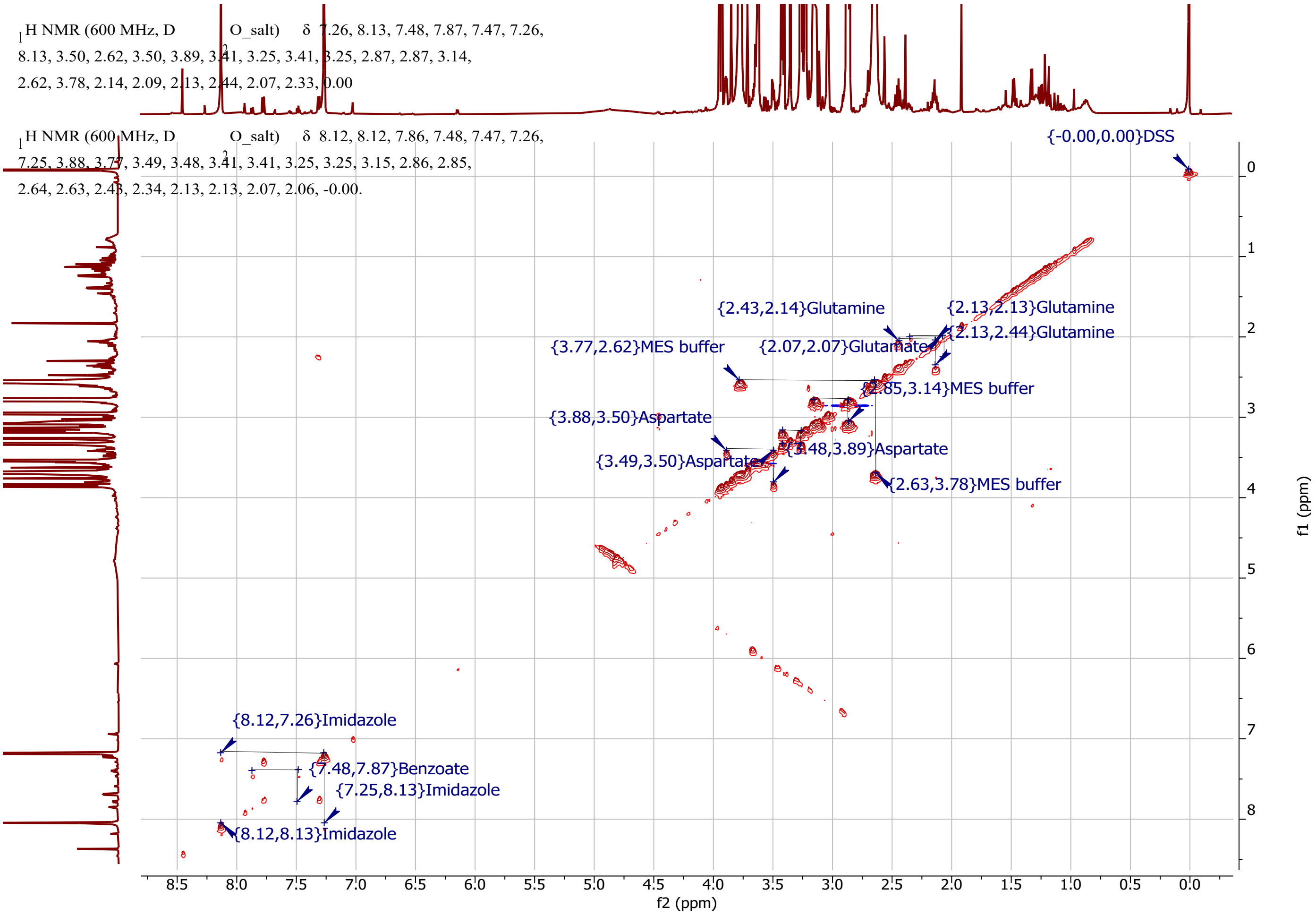

Figure S3. A portion of COSY spectrum for the muscle sample (control 6a: aqueous phase) showing individual metabolites.

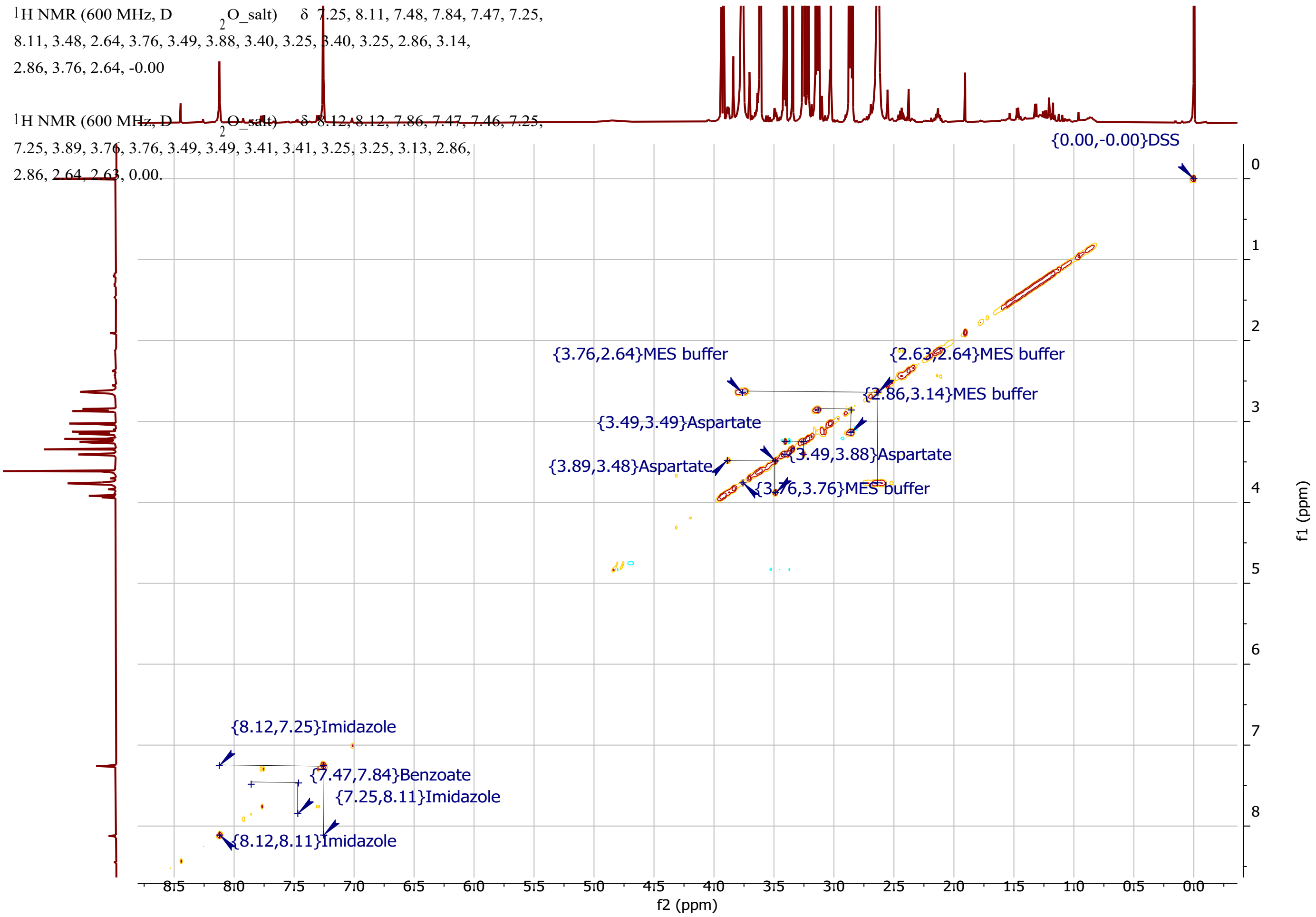

Figure S4. A portion of TOCSY spectrum for the muscle sample (control 6a: aqueous phase) showing individual metabolites.

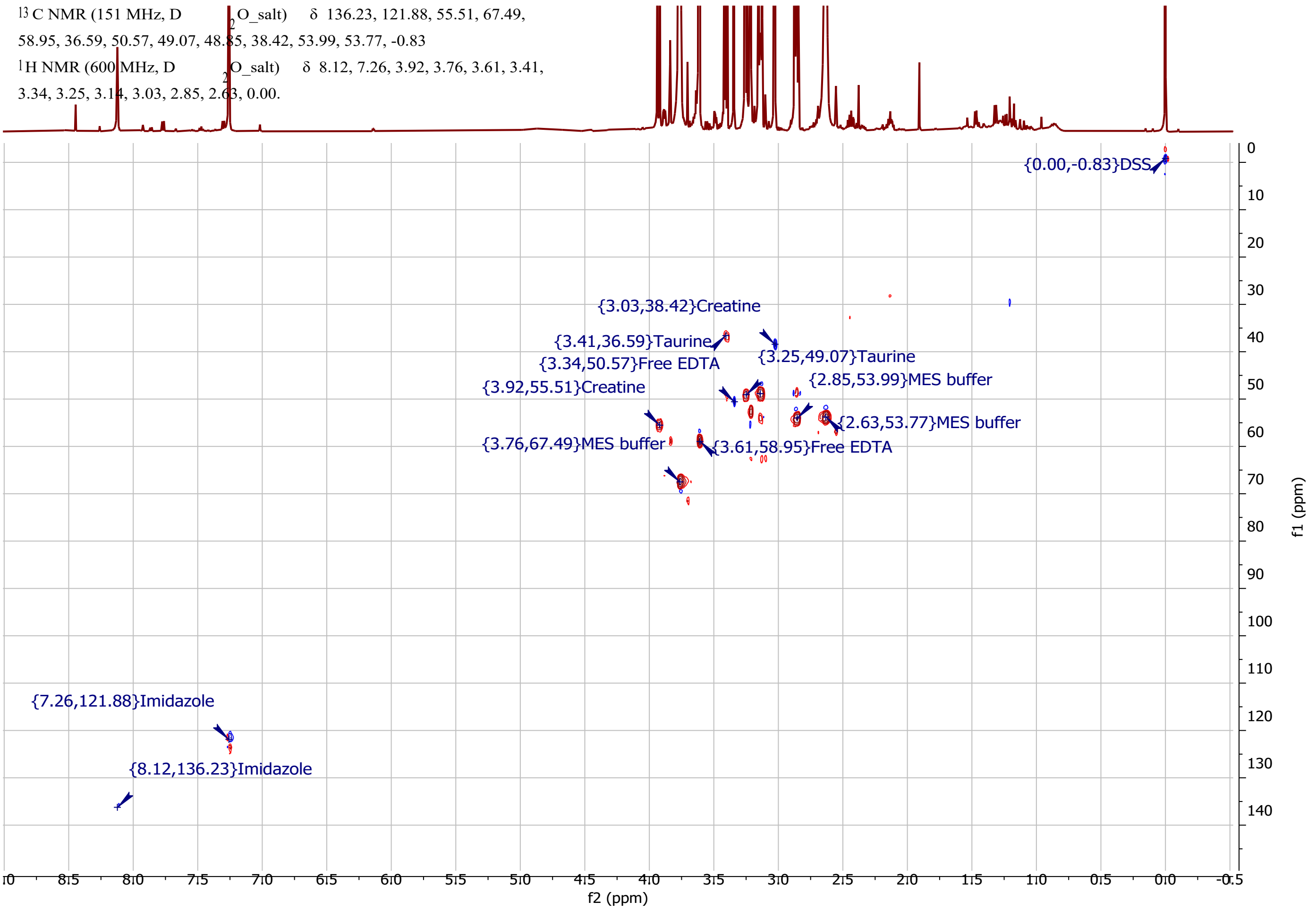

Figure S5. A portion of HSQC spectrum for the muscle sample (control 6a: aqueous phase) showing individual metabolites.

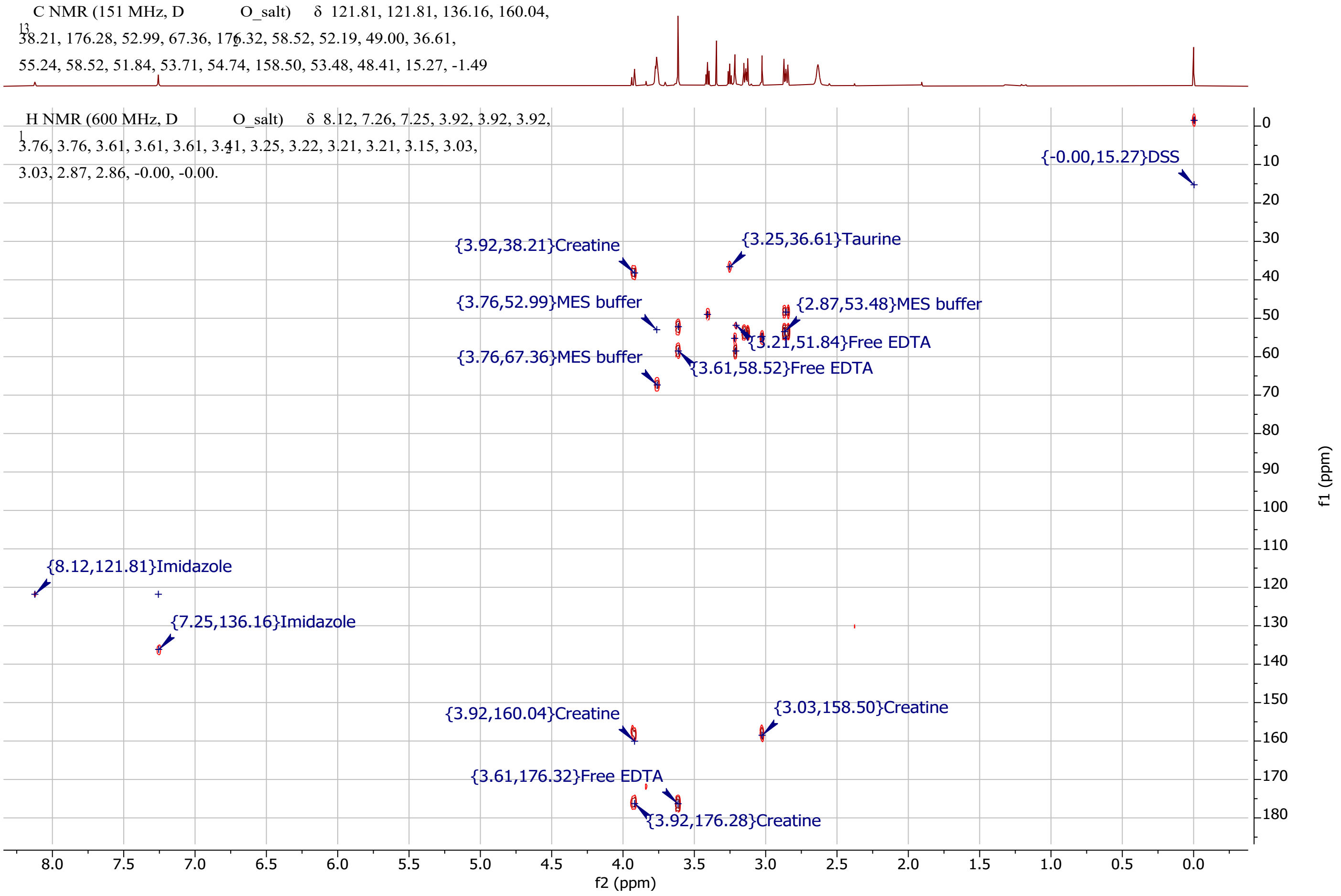

Figure 6. A portion of HMBC spectrum for the muscle sample (control 6a: aqueous phase) showing individual metabolites.
